# Rhabdomyosarcoma cells show an energy producing anabolic metabolic phenotype compared with primary myocytes

**DOI:** 10.1186/1476-4598-7-79

**Published:** 2008-10-21

**Authors:** Teresa WM Fan, Magda Kucia, Kacper Jankowski, Richard M Higashi, Janina Ratajczak, Marius Z Ratajczak, Andrew N Lane

**Affiliations:** 1 Department of Chemistry, University of Louisville, KY, USA; 2 Center for Regulatory Environmental Analytical Metabolomics, University of Louisville, KY, USA; 3 Structural Biology Program, University of Louisville, KY, USA; 4 Stem Cell Biology Program, J.G. Brown Cancer Center, University of Louisville, 529 S. Jackson St., Louisville, KY 40202, USA

## Abstract

**Background:**

The functional status of a cell is expressed in its metabolic activity. We have applied stable isotope tracing methods to determine the differences in metabolic pathways in proliferating Rhabdomysarcoma cells (Rh30) and human primary myocytes in culture. Uniformly ^13^C-labeled glucose was used as a source molecule to follow the incorporation of ^13^C into more than 40 marker metabolites using NMR and GC-MS. These include metabolites that report on the activity of glycolysis, Krebs' cycle, pentose phosphate pathway and pyrimidine biosynthesis.

**Results:**

The Rh30 cells proliferated faster than the myocytes. Major differences in flux through glycolysis were evident from incorporation of label into secreted lactate, which accounts for a substantial fraction of the glucose carbon utilized by the cells. Krebs' cycle activity as determined by ^13^C isotopomer distributions in glutamate, aspartate, malate and pyrimidine rings was considerably higher in the cancer cells than in the primary myocytes. Large differences were also evident in de novo biosynthesis of riboses in the free nucleotide pools, as well as entry of glucose carbon into the pyrimidine rings in the free nucleotide pool. Specific labeling patterns in these metabolites show the increased importance of anaplerotic reactions in the cancer cells to maintain the high demand for anabolic and energy metabolism compared with the slower growing primary myocytes. Serum-stimulated Rh30 cells showed higher degrees of labeling than serum starved cells, but they retained their characteristic anabolic metabolism profile. The myocytes showed evidence of de novo synthesis of glycogen, which was absent in the Rh30 cells.

**Conclusion:**

The specific ^13^C isotopomer patterns showed that the major difference between the transformed and the primary cells is the shift from energy and maintenance metabolism in the myocytes toward increased energy and anabolic metabolism for proliferation in the Rh30 cells. The data further show that the mitochondria remain functional in Krebs' cycle activity and respiratory electron transfer that enables continued accelerated glycolysis. This may be a common adaptive strategy in cancer cells.

## Background

Rhabdomyosarcomas are a serious childhood cancer that arise from a primitive muscle cell, the "rhabdomyoblast", and fail to differentiate into striated muscle cells. These tumors account for about 5–8% of all childhood cancers, with a peak incidence in the 1–5 age group. Overall, 50% of the children diagnosed with rhabdomyosarcoma survive 5 years. There are two main forms of the sarcoma, alveolar and the less severe embryonic form. Although there is considerable information about the origin and progression of these tumors [1-5], much less is known about the biochemical phenotype of these sarcomas and how it differs from the primary myocytes of the nearby tissue. Such information would be of great value not only for understanding the basic biochemistry of these transformed cells, but also for developing new therapeutics. Here we report a study of the biochemical phenotype of rhabdomyosarcoma cells of the embryonic variety, Rh30, in comparison with primary myocytes, using a ^13^C-isotopomer-based metabolomic approach.

Rapidly dividing cancer cells in culture show numerous differences from the differentiated primary cells of a comparable type [6-8]. In order to divide, the cell must traverse the cell cycle repeatedly, with a concomitant doubling of macromolecular content during the S and G2 phases. This requires considerable biosynthetic resources and an energy demand far in excess of the basal rate. Biosynthesis of proteins, nucleic acids, lipids and complex carbohydrates involves coupling to the hydrolysis of nucleoside triphosphates (e.g. ATP and GTP in protein biosynthesis, CTP and UTP in lipid and carbohydrate biosynthesis), as well as the incorporation of carbon and nitrogen from metabolic precursors. For cells in culture, many of these precursors are readily available in the medium, including essential amino acids, fatty acids and glucose. In contrast, the precursors of nucleotides, complex carbohydrates and phospholipids are absent from the medium, and so must be synthesized de novo.

There is a hierarchy of ATP-consuming reactions in cellular function [9]. As might be expected, maintenance of ion gradients across membranes for transport and various macromolecular repair processes involved in cell survival are the highest priority reactions and account for a large fraction of the ATP consumed in quiescent (G0/G1) cells [9-11]. Upregulating macromolecule biosynthesis in preparation for cell division requires additional ATP equivalents to be made. Many cancer cells have a tendency to increase glucose uptake [12] and glycolytic flux even under aerobic conditions, while secreting a large fraction of the glucose carbon as lactate [13,14]. This implies a bypass to oxidative phosphorylation, such that glycolysis alone may account for a substantial fraction of the ATP production. This enhanced aerobic glycolysis is known as the Warburg effect [15-17]. A more efficient means for energy production under aerobic conditions is to increase the flux of acetylCoA derived from the oxidation of glucose, amino acids and fatty acids into the Krebs' cycle. Moreover, to sustain the Krebs cycle activity, it is necessary to upregulate anaplerotic reactions that replenish carbon diverted for biosynthesis.

**Figure 1:**
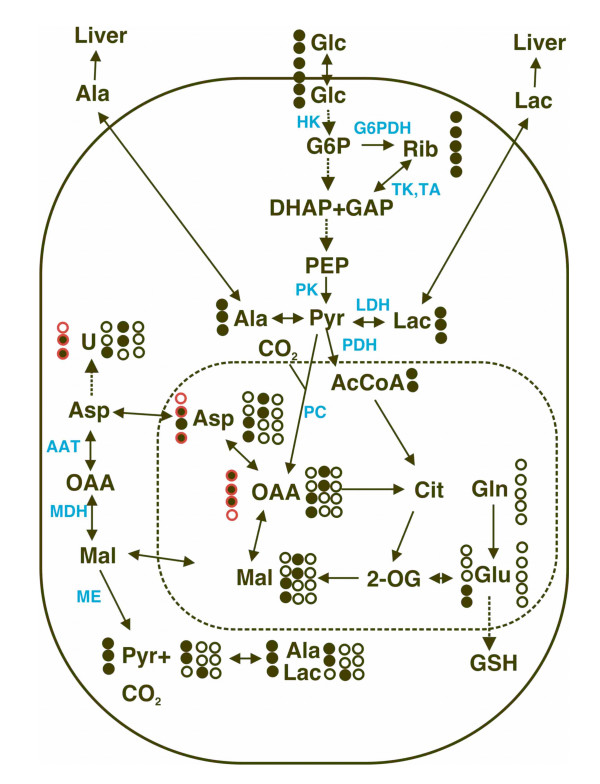
**Metabolic scheme**. Carbon flow from [U-^13^C]-glucose through glycolysis, Krebs cycle, PPP, pyruvate carboxylation, malate/Asp shuttle, and synthesis of GSH and pyrimidine nucleotides as discussed in the text. Also shown are the expected ^13^C isotopomers of key metabolites in this network. Excretion of lactate and Ala into serum is part of the alanine and Cori cycles. Solid oval represents the plasma membrane and the dashed oval represents the mitochondrial space. Double-headed arrows: exchange or reversible processes. The label patterns arise from [U-^13^C]-glucose. Open circles: ^12^C; filled circles: ^13^C. Glc = glucose, G6P = glucose-6-phosphate, Rib = nucleotide ribose, DHAP, GAP = dihydroxyacetone phosphate and glyceraldehyde-3-phosphate, PEP = phosphoenolpyruvate, Pyr = pyruvate, Lac = lactate, Cit = citrate, 2 = OG = 2-oxoglutarate, Mal = malate, OAA = oxalacetate, U = uracil base. Critical enzymes are shown in cyan. HK= hexokinase (entry to glycolysis), G6PDH = glucose-6-phosphate dehydrogenase (entry to the oxidative branch of the pentose phosphate pathway), TK, TA transketolase and transaldolase (non-oxidative pentose phosphate pathway), PK = pyruvate kinase, PDH = pyruvate dehydrogenase, PC = pyruvate carboxylase, AAT = aspartate amino transferase, MDH = malate dehydrogenase, ME = malic enzyme. Glutaminolysis is the pathway from Gln to pyruvate via ME, leading to unlabeled malate, Asp, Ala and lactate. Where two patterns are shown in the Krebs' cycle intermediates, this is due to the scrambling at the succinate step (first turn only). For U and OAA, the labeling from PC activity gives rise to a third labeling pattern, shown in red. The isotopomer pattern for U shows the three ring carbons (4,5,6) derived from Asp. The exchange of [^13^C-1,2,3]-Asp via the malate/Asp shuttle into the cytosol leads to the synthesis of [^13^C-1,2,3]-malate.

Skeletal muscle uses both glycolysis and oxidative phosphorylation for providing energy for contraction. Under conditions of hard exercise, glycogen stores are rapidly depleted, and oxidative phosphorylation of fuels such as fatty acids cannot keep up with demand. The muscle resorts to a greatly increased rate of glycolysis (> 100 fold [18]) and the production of lactate. Lactate, and to some extent Ala, is exported from the myocytes into the blood, when they are transported the liver for resynthesis into glucose via gluconeogenesis (cf. Figure 1). These comprise the Cori and alanine cycles [19,20]. Thus, myocytes have the capacity to make and secrete large amounts of lactate. The secretion of lactate via the monocarboxylate transporter is often accompanied by a proton symport [21,22]. Moreover, most cells can actively export protons via various exchangers, such as the H^+^/Na^+ ^antiporter [23], Many cancer cells pump prodigious quantities of H^+ ^into the extracellular environment, which may offer survival advantages, particularly in the light of their accelerated glycolysis [24,25].

However, it has been reported that many cancer cells upregulate the embryonic M2 form of pyruvate kinase (PK) which is defective in catalyzing pyruvate production [26], so alternative substrates must be oxidized to generate ATP [27-29]. One possibility is to oxidize glutamine/glutamate or serine ultimately to produce pyruvate, bypassing the defective PK step, and allowing lactate production to regenerate NAD^+^[26,29,30]. The relative importance of such reactions is likely to be cell dependent [31,32]. Fortunately, such alternative pathways are readily distinguishable using stable isotope tracer approaches in conjunction with NMR and mass spectrometric analysis, which we have been developing [33-37].

Thus questions arise as to the metabolic differences between a transformed cell and a primary (untransformed) cell. Here we have used ^13^C quantitative isotopomer analysis to follow the fate of individual carbon atoms derived from glucose, as outlined in Figure 1, in both rhabdomyosarcoma cells (Rh30) and primary myocytes, grown under the same conditions to probe their biochemical phenotypes. This approach enabled changes in turnover through individual pathways to be followed, which is not generally possible by concentration-based metabolite profiling alone. Furthermore, the use of ^13^C-edited ^1^H NMR techniques allowed identification of major phosphorylated sugars that are otherwise difficult to detect in crowded ^1^H NMR spectra. Clear differences between the myocytes and the Rh30 cells were evident, consistent with heightened energy and anabolic metabolism in Rh30 cells.

## Results and discussion

### Cell growth

Under the growth conditions of these experiments, the Rh30 cells double in about 24 h in the presence of serum. The growth rate in the absence of serum (0.5% BSA) was about 30% slower, indicating only a weak dependence on growth factors. In contrast, the primary myocytes grew much more slowly, with a doubling time of approximately 50 h in the presence of serum growth factors. This suggests that the cancer cells have an overall net higher demand for metabolic energy to drive macromolecular biosynthesis as the cells proliferate. This is in addition to the normal basal cell metabolic requirements.

### Glucose uptake and lactate secretion

The amount of glucose remaining in the medium and the amounts of lactate and Ala that were secreted by the cells are readily measured by ^1^H NMR and/or GC-MS. Furthermore, as NMR can distinguish between ^13^C-labeled and ^12^C lactate and glucose (see below), it was straightforward to determine the fraction of lactate that derived from the supplied ^13^C-glucose, or from other sources, according to Eq. (2). Table 1 shows the data for glucose consumption and secretion of lactate and Ala into the medium from the two cell types.

**Table 1:** Quantification of labeled metabolites in the media by ^1^H NMR analysis.

Cell	[^13^C Lac] mM	%^13^C_Lac_	[^13^C Ala] mM	%^13^C_Ala_	[Thr] mM	[Glc] mM	Δ[Glc]^a ^mM	%Glc->Lac^b^
Rh30 (24 h)	4.8 ± 0.6	83 ± 1	0.35 ± .03	67 ± 4	0.15	6.1 ± 0.6	4.6 ± .6	52 ± 7.5
Myocyte(48 h)	1.2 ± 0.2	64 ± 2	<0.01	<5	0.15	4.8 ± .5	5.9 ± .5	10 ± 1.4

Concentrations and ^13^C enrichments of selected metabolites in the medium were determined by NMR integrations as described in the Methods.

a change in medium glucose concentration (= consumption).

b percentage of glucose consumed converted to secreted lactate (cf. text).

The consumption of [U-^13^C]-glucose by the Rh30 cells at 24 h was comparable to the consumption by the primary myocytes in 48 h, reflecting the slower growth rate of the latter. The amount and degree of ^13^C enrichment in lactate secreted was very much higher for the Rh30 cells than the primary myocytes (Table 1 and Figure 4). Since the dry weight of the myocytes at 48 h was comparable to that of the Rh30 cells at 24 h, this implies an 8-fold higher lactate production rate in the Rh30 cells than the myocytes. The amount and degree of ^13^C enrichment of Ala in the Rh30 medium was also much higher than measured in the myocyte medium, although Ala secretion constituted a smaller fraction of the labeled glucose consumption (Table 1 and Figure 4). Thus, the Rh30 cells converted a much higher fraction, F (cf. Eq. 2 and Table 1) of the ^13^C glucose consumed into lactate (50%) than the myocytes (10%). We have observed that typically 30–50% of the glucose consumed was converted to lactate by various epithelial derived cancer cell lines, (A.N. Lane, T. WM Fan, unpublished data). This is in contrast to a recent report on glioblastoma cells, in which more than 95% of the glucose consumed was converted to lactate and Ala [38]. The high rate of production of labeled lactate in Rh30 and other cancer cells indicates that the pyruvate kinase (PK) step in glycolysis (Fig. 1) must be active, in contrast with the reports of defective PK in some tumor cells [28].

The high degree of lactate labeling in Rh30 cells indicates that the major source of carbon in lactate and Ala was indeed glucose. However, the fraction of ^13^C Ala labeling in the myocyte was essentially at natural abundance, reflecting its primary origin from the 10% FCS present in the medium. Thus, not only did the Ala labeling pattern in myocytes differ from that in Rh30 cells but also it was distinct from the lactate labeling pattern in both cell lines. As the immediate precursor to Ala and lactate is pyruvate, these differences in labeling patterns could indicate the presence of distinct intracellular pools of pyruvate as observed in other cells [39,40] and see below. Moreover, a significant fraction of glucose taken up by Rh30 (48%) and myocytes (90%) was converted to metabolites other than lactate (cf. Table 1). The fate of some of the remaining glucose carbon was traced in the cellular components is described below

### Glucose-dependent cell metabolism

**Figure 2:**
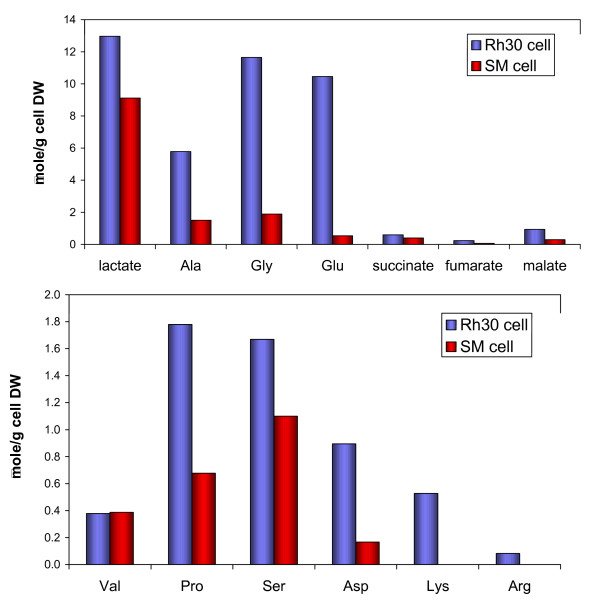
**Concentration of metabolites in cell extracts of Rh30 cells and myocytes**. Concentrations were determined by GC-MS as described in the text. A, B: Total concentrations of selected metabolites normalized to cell dry weight.

**Figure 3:**
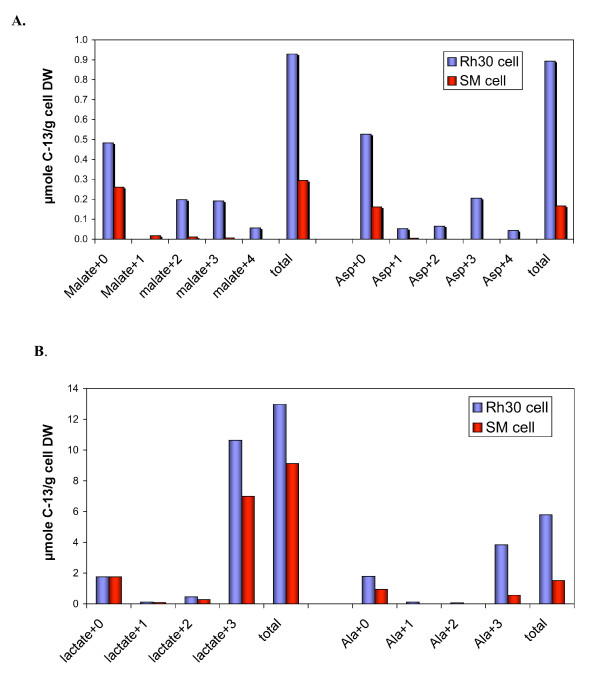
**Concentration of metabolites in cell extracts of Rh30 cells and myocytes**. Concentrations were determined by GC-MS as described in the text. A, B: mass isotopomer concentrations of selected metabolites showing increased labeled incorporation in Rh30 cells.

**Figure 4:**
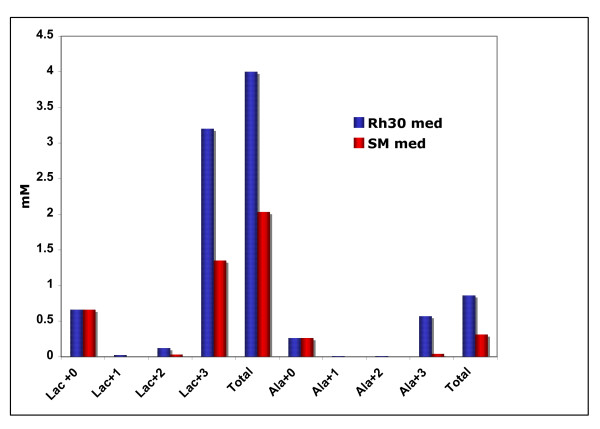
**Concentration of metabolites in media from Rh30 cells and myocytes**. Concentrations were determined by GC-MS as described in the text. Mass isotopomer concentrations of lactate and Ala secreted by Rh30 cells or myocytes into the medium.

**Figure 5:**
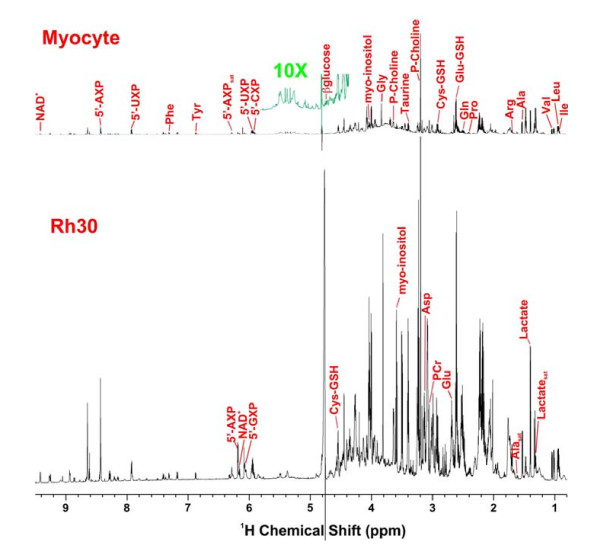
**^1^H NMR spectra of Rh30 and myocyte cell extracts**. NMR spectra were recorded at 600 MHz, 20°C with a recycle time of 5 sec. Cells were grown in the presence 0.2% [U-^13^C]-glucose for 24 h (Rh30) or 48 h (myocytes). Upper myocytes, lower Rh30. Spectra were scaled to dry weight to show the difference in absolute concentrations of metabolites

**Figure 6:**
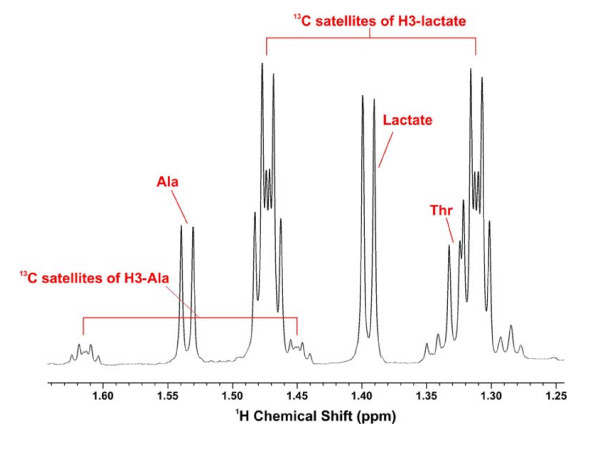
**Alanine and Lactate isotopomer patterns in Rh30 cell extract**. ^1^H NMR spectrum expansion of the Rh30 cell extract spectrum from Figure 5 showing the satellite peaks of lactate and Ala.

Thirty four metabolites in the TCA extracts of the cell biomass were analyzed using GC-MS (Figures 2, 3), twenty of which were also analyzed by ^1^H NMR (Figure 5, 6). An additional eight compounds were determined by ^1^H NMR alone for a total of forty two identified metabolites. The profiles of selected metabolites in Rh30 and myocytes determined by GC-MS are compared in Figures 2 and 3. Lactate, Gly, Thr, Glu, glutathione (GSH), Arg, and phosphocholine (P-choline), taurine and myo-inositol were among the more abundant metabolites (cf. Figures 2, 3, 4, 5). There was good agreement between GC-MS and NMR determinations, indicating high analytical reliability. Figs 2, 3, 4, 5 also illustrate that there are significant differences in levels of numerous metabolites between the myocytes and Rh30 cells. Notably there was a much higher content of amino acids, GSH, taurine, P-choline, myo-inositol, adenine nucleotides (5'-AXP), and uracil nucleotides in the Rh30 cells. The high abundance of P-choline in Rh30 compared with the myocytes (Figure 5) appears to be a distinct feature of proliferating cells [35,37,41-46].

### Glycolysis

**Figure 7:**
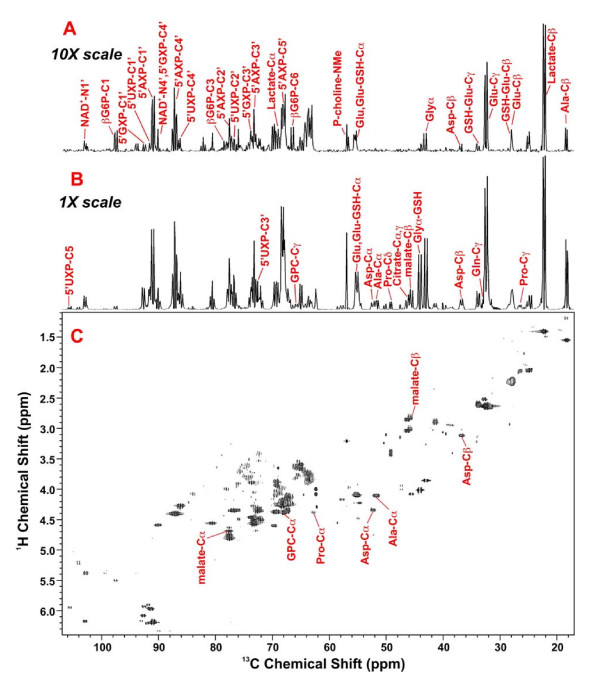
**2-D ^1^H-^13^C HSQC analysis of Rh30 cells and myocytes**. Both cells were cultured in [U-^13^C]-glucose for 24 hr before trichloroacetic acid extraction and HSQC measurement at 14.4 T. The spectra were recorded with 0.14 s acquisition in t_2 _and 34 ms in t_1_. The data table in t_1 _was linear predicted to 1024 complex points and zero filled to 2048, so that ^13^C-^13^C couplings (35–45 Hz) were resolved. Panel C displays the 2-D contour map of the Rh30 cell extract while panels B and A are respectively the 1-D ^13^C projection spectra of the 2-D contour maps for Rh30 and myocytes. Both A and B were normalized to cell dry weight but A was plotted at 10× scale relative to B.

**Figure 8:**
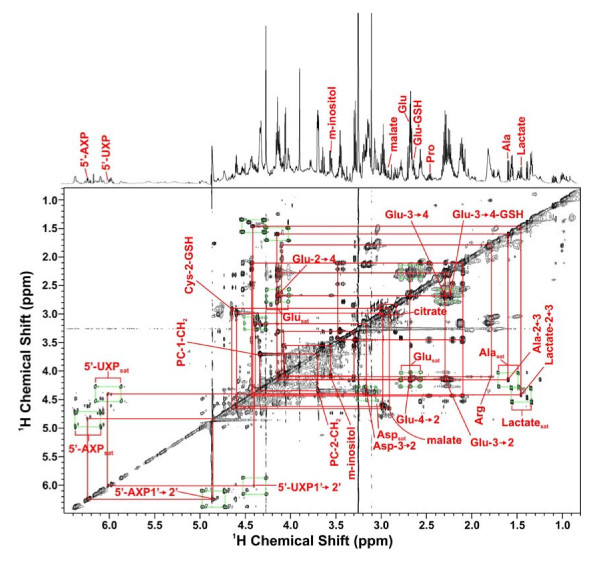
**TOCSY Spectra of Rh30 cell extract**. TOCSY spectrum of TCA extract of Rh30 cells grown for 24 h in the presence of 0.2% [U-^13^C]-glucose and 10% FCS, using a mixing time of 50 ms and a spin lock strength of 8 kHz. Aliphatic region of the spectrum showing connectivities and satellite peaks for some assigned metabolites.

Figure 6 shows an expansion of the lactate region for the Rh30 cells shown in Fig 5. The 3-methyl resonances of both Ala and lactate (doublets at 1.53 and 1.4 ppm, respectively) display pairs of ^13^C satellite peaks displaced 126 Hz apart. These satellite peaks have a complex structure, which can be accounted for by considering additional couplings of these protons to ^13^C2 and ^13^C1 of the compounds (i.e. uniformly labeled) [35,47]. The presence of uniformly ^13^C-labeled lactate and Ala was also evident in the high-resolution 2-D ^1^H-^13^C HSQC or the 1-D ^13^C projection spectra of Rh30 and myocyte cell extracts, as illustrated in Figure 7. The respective doublet and triplet patterns of the β-(C3) and α-(C2) carbons of lactate and Ala (due to ^13^C-^13^C coupling) [36] is further consistent with the ^1^H TOCSY satellite patterns for these two metabolites (cf. Fig. 8). The NMR data were corroborated by the GC-MS analysis, where the m+3 (lactate+3) mass isotopomers predominated. These data together indicate that the three ^13^C in both lactate and Ala derived directly from the [U-^13^C]-glucose precursor via glycolysis.

Detailed quantification of the ^13^C-isotopomers of intracellular lactate and Ala by NMR and GC-MS is given in Table 2 and Fig. 3. As observed for the medium (see above), the myocytes showed a lower degree of enrichment than the Rh30 cells, especially for Ala, consistent with a substantially lower glycolytic rate in the primary cells. The ^13^C-Ala enrichment in the Rh30 cells grown in the absence of growth factors from FCS was even lower than that in the myocytes. There was no unlabeled Ala present in this medium indicating that the cells under growth factor depleted conditions were not actively transaminating labeled pyruvate (cf. Figure 1). Thus, the unlabeled Ala fraction may reflect contributions from other metabolic processes such as protein turnover or glutaminolysis [30] (cf. Figure 1) and possibly involving a different pool of pyruvate as has been observed in other cells [39,40]. However, the amount of protein turnover or glutaminolysis required to account for the production of unlabeled Ala was small owing to its low concentration (cf. Figures 4, 5).

**Table 2:** Quantification of ^13^C enrichment in cellular metabolites by ^1^H NMR analysis: Glycolysis.

Condition	%^13^CAla	%^13^C Lac	^13^CAla/Thr	^13^CAla/Thr
Rh30 (FCS)	74 ± 2	86 ± 2	2.9 ± 0.3	6.7 ± 0.3
Rh30 (BSA)	<5	73 ± 2	<0.1	3.2 ± 0.3
Myocytes	44 ± 2	82 ± 2	0.7 ± 0.1	6.8 ± 0.3

%^13^C was calculated as described in the methods.

### Krebs' cycle activity

**Figure 9:**
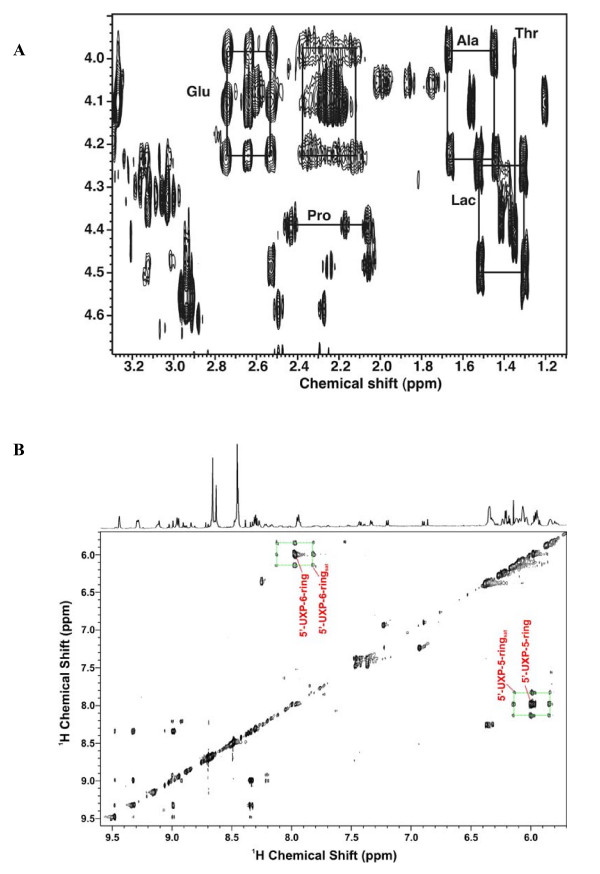
**TOCSY Spectra of Rh30 cell extract**. Expansion of the spectrum shown in Figure 8. A. Glu/Ala/Lac region expansion from A. The boxes show the ^13^C satellite peaks for Ala, Lac and Glu and the absence of labeling in Pro and Thr. Ala and lactate show only fully unlabeled and fully labeled cross peak patterns, whereas the Glu cross peak patterns exhibit both singly and doubly labeled isotopomer species (cf. [35]. B. The partial TOCSY spectral region displays the cross-peak patterns of singly and doubly labeled species for the 5- and 6-ring protons of 5'UXP.

Several other critical metabolites in the cells were also labeled with ^13^C as shown by the 2-D ^1^H TOCSY spectrum of an example Rh30 cell extract in Figure 8. The cross-peak patterns are characteristic of particular metabolites, such as the ribose moieties of nucleotides, lactate, Ala and Glu, as annotated. Many of these cross-peaks are surrounded by satellite peaks that denote the presence of ^13^C. These are made clearer in the expansion of the spectral region of Glu, Ala and Lac (Fig. 9). It is clear that Glu was substantially labeled at the C4 (2.65 ppm) and C2 (4.08 ppm) positions and to a lesser extent at the C3 (2.22 ppm) position. Figure 9 also revealed the presence of four different Glu isotopomers, i.e. ^13^C-2,3-Glu, ^13^C-2-Glu, ^13^C-3-Glu, ^13^C-2,4-Glu, ^13^C-4-Glu, and ^13^C-3,4-Glu. Additional isotopomer information was obtained from analysis of the HSQC spectrum (Fig. 7). The doublet pattern of the γ- (C-4) and α- (C-2) carbons of Glu suggests the presence of ^13^C-4,5-Glu and/or ^13^C-3,4-Glu as well as ^13^C-1,2-Glu and/or ^13^C-2,3-Glu isotopomers, respectively. However, the ^13^C satellite pattern for the TOCSY cross-peak of H3 to H2 of Glu (2.22 to 4.08 ppm in Fig. 9) shows a significant amount of ^13^C-2,3-Glu. Similarly, the dominance of ^13^C-3,4-Glu is clear from the ^13^C satellite pattern for the cross peak of H4 to H3 of Glu (2.65 to 4.08 ppm in Fig. 9) but the presence of ^13^C-4,5-Glu or ^13^C-1,2-Glu could not be ruled out with the present data.

**Figure 10:**
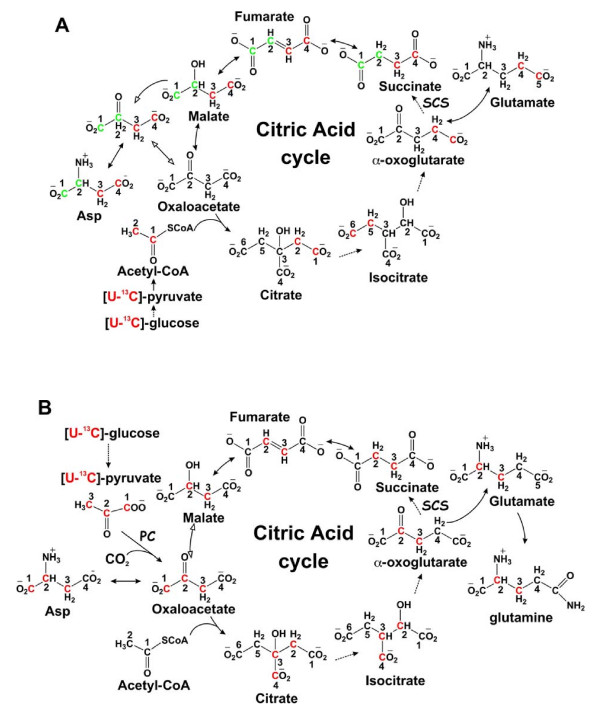
**^13^C labeling patterns in products of glycolysis, PPP and mitochondrial Krebs cycle with [U-^13^C]-Glc as tracer**. The Krebs' cycle reactions are depicted without (panel A) or with (panel B) anaplerotic pyruvate carboxylation (PC) and the labeling patterns illustrated represent one turn of cycle activity. Two distinct ^13^C labeling patterns in Glu result from the cycle reactions with (panel B) or without PC (panel A), i.e. ^13^C labeling at C2,3 or C4,5, respectively. Isotopic scrambling occurs at the symmetric succinate, leading to the redistribution of ^13^C labels into C1 and C2 or C3 and C4 (panel A). Also depicted in panel B is the malate/Asp shuttle across the mitochondrial membrane, where the export of [^13^C-1,2,3]-Asp into the cytosol leads to the synthesis of [^13^C-1,2,3]-malate. Red C and green C represent ^13^C labeled carbons; solid and dashed arrows denote single and multi-step reactions, respectively; open arrows in panel A delineate unlabeled starting oxaloacetate (OAA) from ^13^C-labeled OAA after one turn; open arrow in panel B denotes the start of a second cycle; double headed arrows indicate reversible or exchange reactions; SCS denotes succinyl CoA synthetase.

The above results are clear evidence of de novo synthesis of Glu from labeled glucose (10 mM), even though unlabeled Gln (2 mM) and Glu (0.13 mM) were both present in the medium. Further examination of the labeling pattern revealed evidence for the route of synthesis for the different Glu isotopomers. Glutamate is derives from 2-oxoglutarate by transamination or reductive amination (Glu dehydrogenase) in mammalian cells. There are two routes of label incorporation from [^13^C-U]-glucose into 2-oxoglutarate via pyruvate (Figures 1, 10). As shown in detail in Figure 10, one route (Fig. 10A) involves the pathway pyruvate → acetylCoA → citrate → 2-oxoglutarate, which leads to the production of ^13^C-4,5-Glu. The other route (Fig. 10B) utilizes the anaplerotic reaction of pyruvate carboxylase to produce OAA directly from pyruvate with the subsequent reaction sequence of citrate → 2-oxoglutarate, which generates ^13^C-2,3-Glu. The presence of ^13^C-2,3-Glu shown in Figures 7 and 9 is consistent with the activity of pyruvate carboxylase. The redistribution of labels into different carbons of Glu is presumably the result of carbon scrambling at the succinate dehydrogenase (SDH) step due to the symmetry of the succinate molecule. Moreover, the presence of multiple ^13^C-Glu isotopomers described above supports multiple turns of the cycle with ^13^C label input (cf. Figure 10).

It is clear from Figure 7 that the Rh30 cells had a much higher buildup of the different ^13^C isotopomers of Glu (than the myocytes. This implicates a higher turnover of the Krebs cycle and specifically a higher capacity for anaplerotic pyruvate carboxylation, as evident by the enhanced buildup of ^13^C-4,5-Glu and ^13^C-2,3-Glu, respectively.

Malate and aspartate also showed substantial ^13^C enrichment (Figure 3B) in both doubly and triply labeled mass isotopomers based on the GC-MS analysis. The positional isotopomer of the triply labeled malate was deduced from the 2-D HSQC analysis, as shown in Figure 7. The doublet of C3 or β-malate and the triplet of C2 or α-malate indicate the prevalence of [^13^C-1,2,3]-malate. The doubly labeled malate isotopomers can be [^13^C-2,3]-malate, [^13^C-1,2]-malate, and/or [^13^C-3,4]-malate, the abundance of which could not be ascertained with the present data. By comparing Rh30 cells with myocytes in terms of the ^13^C-malate isotopomer patterns in Fig. 3B and 7, it is clear that Rh30 cells synthesized a much higher amount of doubly and triply labeled malate, the latter of which was identified as [^13^C-1,2,3]-malate. Similar considerations apply to Asp i.e. the doubly and triply labeled ([^13^C-1,2,3]-Asp) isotopomers were more abundant in Rh30 cells than in myocytes (Figure 3B).

According to the schemes in Figs 1 and 10B, [^13^C-1,2,3]-malate and [^13^C-1,2,3]-Asp can only be synthesized from [U-^13^C]-pyruvate via anaplerotic pyruvate carboxylation (PC). PC can also lead to the synthesis of the doubly labeled [^13^C-2,3]-malate or Asp after one turn of the Krebs cycle, as outlined in Fig. 10B. In contrast, the doubly labeled malate species (as [^13^C-1,2]- and [^13^C-3,4]-malate) or Asp should derive from the normal Krebs' cycle sequence with input of [U-^13^C]-pyruvate, as illustrated in Fig. 10A, or the doubly labeled [^13^C-2,3]-malate from pyruvate carboxylation, as outlined in Fig. 10B. Thus, the combined GC-MS and NMR determination of malate and Asp isotopomers not only revealed an enhanced activity of the Krebs' cycle but also activation of pyruvate carboxylation in the Rh30 cells over the primary myocytes. Such anaplerotic reactions are essential for actively dividing cells, as the production of important metabolites such as pyrimidine nucleobases and reduced glutathione deplete Krebs' cycle intermediates (Fig. 1 and see below), which must be replenished.

Krebs cycle intermediates may also be replenished via glutaminolysis [30,38,48], which has been shown to be activated in glioblastoma cells, based on the production of labeled lactate derived from labeled Gln [38]. The role of glutaminolysis in anaplerosis in Rh30 cells could not be ascertained here for lack of carbon tracing from labeled Gln. However, the comparable production of unlabeled lactate by Rh30 cells and myocytes (Figures 1, 4 and see above) suggests a minor role for glutaminolysis in activating anaplerosis in Rh30 cells.

The sustained activity of the Krebs' cycle in the Rh30 cells requires a functional respiratory chain to regenerate NAD^+ ^and FAD in the mitochondria. The same chain also reoxidizes cytosolic NADH produced by glycolysis, and transported into the mitochondria via the malate-aspartate shuttle (cf. Fig. 1), that is not involved in reducing pyruvate to lactate (and see above).

Furthermore, transformed Rh30 cells were much more active in de novo GSH synthesis than the primary myocytes. GSH was labeled in the Glu residue in Rh30 cells and the extent of this labeling was much stronger in Rh30 cells than in the myocytes (cf. Figs. 7 and 8), just as the case for its precursor Glu (Table 4). Although the cysteine supply can be rate limiting in GSH synthesis, in this case the medium contained an adequate supply of cystine (0.2 mM). The incorporation of labeled glutamate indicates a substantial rate of de novo GSH synthesis in the Rh30 cells.

### Nucleotide biosynthesis

Asp is a direct crucial precursor to pyrimidine biosynthesis (Fig. 1). We have previously shown that the pyrimidine rings of nucleotides become ^13^C labeled in the Rh30 cells [35]. All possible isotopomers of 5'-UXP C5C6 were observed as shown in the TOCSY spectra (Figure 9B). This ^13^C satellite pattern was similar to that of Asp (Figure 8), which is consistent with Asp being a direct precursor to the de novo synthesis of 5'UXP ring (Figures 1, and 10B). The ^13^C abundance and extent of the labeling in uracil ring of 5'-UXP (Table 3) and its precursor Asp (Figures 3B and 7). was much higher in Rh30 cells than in myocytes. Thus, it is clear that Rh30 cells exhibited a higher capacity for the *de novo *synthesis of pyrimidine nucleotides than myocytes.

**Table 3:** Quantification of ^13^C enrichment in cellular metabolites by ^1^H NMR analysis: Pentose phosphate and pyrimidine biosynthesis

	Ribose ring		Base ring
Condition	%^13^C Pur	%^13^C Pyr	[%^13^C Uri

Rh30 (FCS)	95 ± 2	80 ± 2	50 ± 3(26 ± 2)^a^
Rh30 (BSA)	82 ± 2	88 ± 2	45 ± 2(21 ± 2)^a^
Myocytes	>90	<5	<5

a All 4 possible isotopomers at the C5, C6 positions were observed. first entry: percentage of all forms of ^13^C5 and ^13^C6 of U. The value in parenthesis the percentage of doubly labeled C5 and C6.

**Table 4:** Quantification of ^13^C enrichment in cellular glutamate by ^1^H NMR analysis.

			% ^12^C or	^13^C				
Condition	^12^α^12^γ	^12^α^13^γ	^13^α^12^γ	^13^α^13^γ	^12^α^12^β	^12^α^13^β	^13^α^12^β	^13^α^13^β

Rh30 (FCS)	46 ± 3	11 ± 2	14 ± 2	29 ± 3	63 ± 3	2.5 ± .5	6.7 ± 1	28 ± 2
Rh30 (BSA)	54 ± 3	16 ± 2	<2	30 ± 3	78 ± 4	<2	9 ± 1	13 ± 1
Myocytes	71 ± 3	9 ± 1	12 ± 2	7 ± 1	>90	<10	<2	<2

As Asp is derived from the transamination of the Krebs' cycle intermediate, OAA, increased diversion of Asp to pyrimidine ring biosynthesis would ultimately deplete OAA, which must be replenished to sustain the Krebs' cycle activity. As described above, this can occur by enhanced anaplerosis via pyruvate carboxylation in Rh30 cells. Furthermore, labeled Asp synthesized in the mitochondria is presumably transported to the cytoplasm for pyrimidine biosynthesis via the Asp/malate shuttle (Fig. 1). The enhanced rate of pyrimidine synthesis in Rh30 cells may well reflect an increased Asp/malate shuttle activity. This shuttle also facilitates the transfer of NADH produced in the cytoplasm to the mitochondrion for ATP production via oxidative phosphorylation. Although pyrimidine biosynthesis is mainly cytoplasmic, one step occurs within the mitochondrion and involves coupling to the electron transfer chain. Collectively these all point to metabolically functional mitochondria in the cancer cells.

The higher capacity for pyrimidine synthesis in Rh30 cells is corroborated by the^13^C labeling data in the ribose moiety of 5'-UXP, where all five carbons of the ribose residue were uniformly labeled in ^13^C. This was evident from the TOCSY ^13^C satellite patterns of the 5'-UXP cross-peaks (e.g. Fig. 9B for H1' → H2' of 5'-UXP) and ^13^C spin coupling patterns of C1' to C4' of ribose in 5'-UXP (Fig. 8). The abundance of [U-^13^C-ribose]-UXP (Fig. 9B) and % ^13^C enrichment (Table 3) were much higher in Rh30 cells than in myocytes, consistent with an enhanced rate of 5'-UXP synthesis. Also evident in Fig. 8 is the higher rate of synthesis for purine nucleotides (i.e. 5'-AXP and 5'-GXP), as shown by the higher abundance of [U-^13^C-ribose]-5'-AXP and 5'-GXP. This difference in ^13^C labeling of nucleotides is consistent with a higher metabolic demands for macromolecular (e.g. RNA) biosynthesis for the transformed cells compared with slowly dividing primary cells (also see below).

It should also be noted that the ^1^H NMR resonances of 5'-AXP and 5'-UXP in Fig. 8 were in fact mixtures of 3 resonances, which correspond to the tri, di and mono phosphates species, with intensities in the order 5'-ATP > 5'-ADP > 5'-AMP and similarly for 5'-UTP, 5'-UDP and 5'-UMP (data not shown).

### Intracellular glucose-6-phosphate and glycogen synthesis

**Figure 11:**
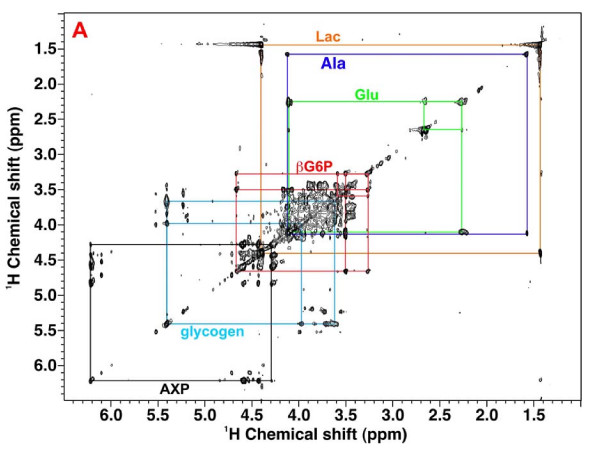
**HCCH TOCSY spectrum of myocyte cell extract**. 48 h myoctye cells and 24 h Rh30 cells grown in the presence of [U-^13^C]-glucose were extracted as described in the methods. 1D NMR spectra were recorded at 18.8 T as described in the methods and elsewhere[35]. The HCCH TOCSY spectrum of the myocyte extract was recorded with a mixing time of 12 ms showing cross peak patterns of consecutively labeled carbons of glycogen resonances, G6P and fully labeled ribose moieties of nucleotides (e.g. 5'AXP), lactate, Ala, and Glu.

Additional 2-D ^1^H NMR experiments helped identify ^13^C-labeled glucose-6-phosphate (G6P) and/or glycogen in the Rh30 and myocyte extracts. These included ^1^H HCCH-TOCSY and ^1^H-^13^C HSQC-TOCSY. The HCCH-TOCSY spectrum of the myocyte extract (Figure 11) displayed covalent connectivities of protons attached to consecutively ^13^C-labeled carbons of various metabolites such as lactate, Ala, Glu, β-glucose-6-phosphate (βG6P), glycogen, and AXP. The Rh30 spectrum (not shown) also exhibited similar covalent networks except for the absence of connectivities for glycogen, lower abundance connectivities for βG6P, and more prominent connectivities for 5'-UXP and 5'-GXP. The connectivity patterns for these metabolites is consistent with the presence of [^13^C-1-5]-βG6P and [U-^13^C-glucose]-glycogen, in addition to [U-^13^C]-lactate, [U-^13^C]-Ala, and [^13^C-2,3,4]-Glu.

**Figure 12:**
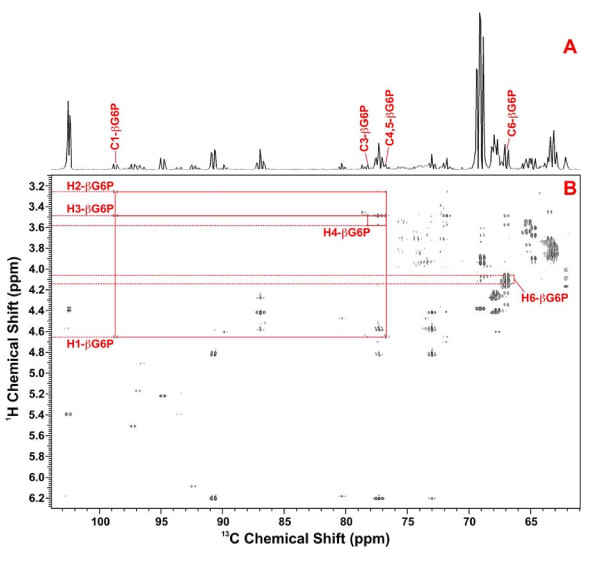
**HSQC TOCSY spectrum of myocyte cell extract**. Sugar region of a high resolution HSQC-TOCSY spectrum of myocytes recorded with a mixing time of 50 ms. The resolution in F1 is adequate to show ^13^C-^13^C splittings in for example the beta form of G6P. upper: 1D projection, lower: 2D spectrum

To confirm further the assignment for G6P (as opposed to glucose), 2-D high-resolution ^1^H-^13^C HSQC-TOCSY spectra were analyzed and that for the myocytes is illustrated in Figure 12. In this spectrum, covalent connectivities from ^13^C1 to H1 then relayed to H2 and H3 as well as ^13^C3 to H3 then to H4 of βG6P were clearly observed. Also evident was the set of ^13^C6 to H6 connectivities of βG6P, thus providing unequivocal identification of βG6P in the crude extract of the myocytes. Moreover, the high-resolution spectrum revealed ^13^C coupling patterns of βG6P carbons, i.e. doublet for C1, triplet for C3-5, as well as doublet for C6, which indicate the presence of uniformly labeled βG6P and thus its origin from [U-^13^C]-glucose.

Free glucose is not expected to be present in cells where the activity of hexokinase exceeds that of glucose transport [49]. The 1D proton spectra (Fig. 5A) show that the concentrations of glucose and G6P were negligible in the Rh30 cells which indicates that the steps downstream of G6P in glycolysis are not flux limiting. The low concentration of free glucose is a feature of cancer cells which together with the low level of G6P reflects the high glycolytic flux, making glucose transport rate limiting [50], despite derepression of GLUT4 expression by mutated p53[12]. In contrast, the concentration of G6P in the myocytes was significant (Fig 7A), which suggests that for these cells, the uptake of glucose or hexokinase was not limiting glycolysis in these cells. Rather, a step downstream of G6P, such as PFK1, limits flux leading to an accumulation of G6P. This is consistent with typical estimates of intracellular G6P in skeletal muscle (ca. 200–400 μM)[51]. The much higher demand from glycolysis and the PPP (see above) in Rh30 cells, compared with the myocytes, may well be limiting glycogen synthesis, which competes for the same glucose precursor. This may underlie the lack of newly synthesized glycogen in Rh30 cells versus its significant presence in the myocytes (cf. Fig. 11).

Finally, the extent of ^13^C labeling in lactate, Ala, and pyrimidine nucleotides was higher in Rh30 cells grown in fetal calf serum (FCS) than cells grown in BSA (Table 3). The low enrichment of the intracellular Ala may be related to differences in pyruvate production from sources other than glucose between the FCS-stimulated and unstimulated Rh30 (BSA) cells (see above). For Glu, the two growth conditions gave a comparable extent of ^13^C labeling, which was significantly higher than that for the myocytes. Thus, even without the influence of the growth factor in FCS, Rh30 cells grown in BSA still exhibited a greater turnover of [U-^13^C]-glucose through glycolysis, Krebs' cycle, and pyrimidine nucleotide biosynthesis than the primary myocytes.

## Conclusion

Using [U-^13^C]-glucose as tracer and isotopomer-based metabolomic analysis, we have demonstrated major distinctions in central energy and anabolic metabolism between the primary myocyte and transformed Rh30 cell lines. A major advantage of this approach is the ability to follow the fate of individual atoms simultaneously into many metabolites, and thereby to characterize the interrelationships among multiple biochemical pathways. Our data show that relative to the myocytes, glycolysis, Krebs' cycle, pentose phosphate pathway, and nucleotide biosynthesis (cf. Fig. 1) were coordinately enhanced in Rh30 cells, presumably to meet the demand of accelerated growth. The coordination between energy production (including the Warburg effect [15,17,52]) and generation of biosynthetic precursors in Rh30 cells may well be mediated via an activation of anaplerotic carboxylation and the malate/Asp shuttle which facilitates the transfer of NADH hydride from the cytoplasm to the mitochondrion for oxidative phosphorylation [7,48], thereby sustaining accelerated glycolysis. The role of glutaminolysis as another anaplerotic input to the Krebs cycle is unclear but is likely to be less significant in accounting for the distinction between Rh30 cells and myocytes.

It is therefore evident that mitochondria of transformed Rh30 cells are active, both in citric acid cycling and respiratory electron transport. Since these processes are essential to cell proliferation, it is reasonable to postulate that they may be fundamental in the transformation of primary to malignant cells.

## Methods

### Materials

#### Cells

The human RMS cell line Rh30 (established at St. Jude Hospital), and human primary myocytes were cultured in pyruvate and glucose-free RPMI 1640 (Invitrogen, 11879-020), supplemented with 100 IU/ml penicillin, 10 μg/ml streptomycin, and 50 μg/ml neomycin (Life Technologies, Inc., Grand Island, NY) in the presence of 10% heat-inactivated FCS (Life Technologies) and 0.2% glucose. The medium thus contained 0.17 mM ^12^C alanine and 0.5 mM unlabeled lactate and 0.56 mM unlabeled glucose from the FCS. The cells were cultured in a humidified atmosphere at 5% CO_2_, 37°C at an initial cell density of 2.5 × 10^4 ^cells/flask (Corning) and the media were changed every 48 h. Prior to ^13^C labeling the cells were grown in 0.5% bovine serum albumin (BSA), without FCS to partially synchronize the culture. Control experiments were also run under otherwise identical conditions in which 0.5% BSA was used in place of FCS during the labeling phase. The dry weight of cells was measured for normalizing metabolite concentrations.

#### Isotopes

[U-^13^C]-glucose (99% ^13^C) was purchased from Cambridge Isotope Laboratories, MA and used without further purification. A stock solution (20%) was prepared in phosphate buffered saline and sterile filtered through a 0.2 μm filter. The appropriate quantity of the stock solution was added to glucose-free RPMI medium to a final concentration of 10.7 mM (excluding the 0.54 mM unlabeled glucose from the FCS).

### Methods

Initial experiments were carried out with unlabeled glucose, followed by full metabolite profile experiments using [U-^13^C]-glucose. In one experiment, both the Rh30 and myocytes were cultured for 24 h in labeled glucose. In a second experiment to account for the differences in cell doubling rates, the Rh30 cells were cultured for 24 h, and the myocytes for 48 h in the labeled glucose. In this work, ^13^C atoms in various metabolites deriving exclusively from [U-^13^C]-glucose were monitored by NMR or mass spectrometry, which represented de novo metabolic transformations originating from glucose. Growth rates and viability were measured by direct cell counting on a hemocytometer using 0.4% Trypan Blue.

### Metabolite extraction

After growth for 24 or 48 h the cells were harvested by trypsinization, scraping followed by low spin centrifugation at 4°C. The pellet was washed twice with ice-cold PBS to remove contaminating medium, pelleted again, flash frozen in lN_2 _and lyophilized. The dry cell mass was recorded for normalizing metabolite concentrations before extractions twice with ice-cold 10% trichloroacetic acid (TCA), followed by lyophilization as previously described[37,53]. The dry mass of the cells was recorded for normalizing metabolite concentrations. The medium was similarly treated with TCA in the same way for measuring secreted metabolites (predominantly lactate and Ala), and to assay the glucose utilization during the experiment. ^13^C-glucose was quantified at t = 0 and at harvest (t) by^1^H NMR using the well-resolved ^13^C1 satellite signals of -α glucose centered at 5.22 ppm. This accounts for 36% of the total glucose. The ^13^C and ^12^C lactate and Ala concentrations were determined by integration of the methyl peak and its satellites and calibrated against the concentration of lactate determined independently by GC-MS. From this, the amount of glucose consumed, ΔGlc, and the fraction converted to lactate plus Ala, F, could be estimated, according to Eq. (1) and Eq. (2) [35]:

(1) ΔGlc = n(^13^C-Glc)^0^-n(^13^C-Glc)^t^]

(2) F = [n(^13^C-lac) + n(^13^CAla)]/2ΔGlc

n is the number of moles of the compound in parentheses.

The factor of 2 accounted for the fact that one molecule of glucose gives rise to two molecules of lactate or Ala.

1-F then represents the glucose carbon that enters other metabolites and macromolecules in the cell mass or otherwise not accounted for. The fractions were not been corrected for the small (5%) contribution from unlabeled glucose present in the medium.

### NMR

NMR spectra were recorded at either 14.1 T or 18.8 T on Varian Inova NMR spectrometers at 20°C using a 90° excitation pulse. One-dimensional NMR spectra were recorded with an acquisition time of 2 sec and a relaxation delay of 3 sec. Under these conditions, the protons were essentially full relaxed, as determined by independent measurements of the spin-lattice relaxation time, T_1_. For identifying metabolites in the extracts and determining the positional enrichment with ^13^C we used a suite of 2D experiments including TOCSY (or DQ COSY), HSQC, HCCH-TOCSY and HSQC-TOCSY as previously described [35-37,53]. The latter two experiments make use of the isotope editing function. HCCH-TOCSY specifically selects for those molecules in which at least two adjacent carbon atoms are ^13^C whereas HSQC or HSQC-TOCSY detects protons directly attached to ^13^C. The TOCSY experiments were recorded with a spectral width of 6000 Hz in F2, 0.341 s acquisition time in t_2 _and 0.05 s in t_1_, 1.9 s interpulse delay, 50 ms mixing time, and an 8 kHz B_1 _field strength. The HSQC-TOCSY experiments were recorded with an acquisition time of 0.12 s in t_2 _and 12 ms in t_1 _using a 50 ms proton spin lock at a strength of 8 kHz. For the HCCH-TOCSY experiments, the acquisition times were 0.12 s in t_2_, 0.05 s in t_1 _using an 8 kHz ^13^C spin lock field centered in the aliphatic region of the spectrum for a duration of 12 ms.

Metabolites were assigned based on their ^1^H chemical shift, TOCSY connectivity patterns and correlation with ^13^C, using our in-house database as described previously [36,54]. All metabolites except choline, sugars and the nucleotides were quantified from GC-MS data [55] whereas choline was quantified from the peak at 3.22 ppm in the 1-D NMR spectra, as described previously [56].

### NMR analysis of ^13^C enrichment

^13^C enrichment in lactate and Ala was determined from 1D ^1^H NMR experiments, as the methyl resonances of these metabolites were well resolved. The peaks were integrated, and the areas of the protons attached to ^12^C (central peak) and to ^13^C (satellite peaks) were recorded. The ^13^C content was then calculated as [35]:

(3) %^13^C = 100*area(^13^C satellites)/[area(^12^C)+area(^13^Csatellites)]

For other metabolites, which were not resolved in 1D NMR experiments, we volume-integrated cross-peaks in TOCSY spectra after correcting the base-planes. The various isotopomer enrichments were calculated as for the 1D spectra, i.e. the peak volume of the particular ^13^C isotopomer divided by the total volume of the cross peak including the satellites. In the TOCSY experiments, the protons were partially saturated owing to the shorter recycle time (2 s) of these experiments. Thus, the actual peak volumes were corrected according to the differential T_1 _values of protons attached to ^13^C or ^13^C; as:

(4) M(true) = M(obs)/[1-exp-t/T_1_]

Where M(true) is the corrected area, M(obs) is the observed area, and t is the recycle time. As the relaxation during the spin lock has the opposite effect on peak intensities, this correction was small [35]. Effective T_1 _values were determined on standards recorded under the same solvent conditions.

### GC-MS

Following NMR analysis, an aliquot (50–100 μl) of the NMR sample was re-equilibrated with H_2_O and lyophilized to remove any deuteration, then silylated with 25–50 μl 1:1 (v/v) acetonitrile:MTBSTFA (N-methyl-N-[tert-butyldimethylsilyl]trifluoroacetamide, (Regis Chemical, Morton Grove, IL) by 3 h of sonication followed by standing overnight [57,58]. The solution was directly analyzed on a PolarisQ GC-ion trap MSn (ThermoFinnigan, Austin, TX) using a 0.5 μl injection volume. The column was 0.15 mm i.d. × 50 m fused silica open-tubular, coated with 0.2 μm BPX-5 (5% phenyl-methylsiloxane). The following conditions were employed for GC-MS. Injector at 280°C, column at 60°C for 2 min, followed by a 20°C/min ramp to 150°C, then 6°C/min to 300°C, splitless vent held for 1.5 min, He carrier gas velocity 30 cm/s at 60°C, transfer line = 280°C, electron energy = 70 eV, source heated to 190°C, automatic gain control target value = 50, maximum inject time = 25 ms, He damping gas = 0.3 ml/min, full scan acquisition from 140 to 650 m/z at a rate of five spectra/s which were averaged into one, and mass calibration preformed byperfluorotributylamine.

Metabolites were identified and quantified automatically using Xcalibur software (ThermoFinnigan), based on their GC retention times and mass fragmentation patterns matched against an in-house database and external standards. Identities were also verified by manual inspection. GC-MS quantification of total abundance of metabolites was accomplished by comparing the m/z 147 ion response for each metabolite in the samples with that for the corresponding standard of known concentration. The m/z 147 ion was chosen for quantification of labeled amino acids because this ion fragment did not contain ^13^C as it originated entirely from the silyl moiety. Conversely, for ^13^C isotopomers, the pseudo-molecular ion clusters of authentic standards were used to obtain the empirical ion ratios as the basis of the calculation for label abundance, as described in detail previously [37].

## Competing interests

The authors declare that they have no competing interests.

## Authors' contributions

MK, KJ, JR, grew cells and determined growth curves; TWMF extracted the cells and recorded and interpreted NMR spectra; RMH acquired and analyzed the mass spectra; MZR directed cell biology and collaboration; ANL recorded and interpreted the spectra. TWMF and ANL drafted the manuscript. All authors have read and approved the contents.
